# Aging and Dementia in Neurological and Medical Disorders: Cognitive, Neurological, and Psychosocial Insights and a Framework for More Inclusive Research

**DOI:** 10.1002/alz70857_104727

**Published:** 2025-12-24

**Authors:** Cristina A.F. Román, Anny Reyes

**Affiliations:** ^1^ Keck School of Medicine of USC, Los Angeles, CA, USA; ^2^ Cleveland Clinic, Cleveland, OH, USA

## Abstract

**Background:**

Individuals with neurological and other medical disorders have largely been excluded from aging and dementia research. In fact, when considering some of the largest, most impactful aging studies, cohorts, and clinical trials, the presence of a neurological/medical disorder is often an exclusion criteria due to concerns of confounding effects. Studies focusing on these groups in the context of aging have offered unique insights into the aging process, however, as well as possible mechanisms that contribute to phenotypic heterogeneity, particularly in Alzheimer's disease. As such, this review study will highlight literature in aging/dementia for several neurological/medical disorders and present a framework for aging/dementia and inclusive research.

**Method:**

Literature reviews were conducted using established scientific search engines (e.g., PubMed, Medline) with a focus on seven neurological/medical disorders, including multiple sclerosis (MS), epilepsy, traumatic brain injury (TBI), Parkinson's disease, Huntington's disease, and central nervous system (CNS) and non‐CNS cancers. Searches were conducted for each disorder separately using keywords related to the disorder, cognition, brain‐related variables (e.g., neuroimaging), dementia, and psychosocial factors (e.g., social determinants of health). Primary exclusion criteria: (1) absence of variables of interest; and (2) reviews, meta‐analyses, dissertations, or abstracts.

**Result:**

Overall, we found that aging/dementia research in many of these groups was limited (e.g., MS, TBI). For several disorders there is evidence for accelerated aging and/or increased risk of dementia. Unique cognitive profiles are present for several groups (e.g., MS, epilepsy), while chronicity appeared to play a role in cognitive outcomes in others (e.g., TBI). Historically minoritized groups are disproportionately impacted by neurological/medical disorders due to systemic and social factors that contribute to poorer aging and greater risk of dementia, further perpetuating health disparities. A framework for aging in neurological/medical disorders and ways to integrate these groups into future research will also be presented.

**Conclusion:**

The current review demonstrates the value of focusing on neurological/medical populations in aging/dementia research, especially with respect to the unique information gained about cognitive, neural, and psychosocial mechanisms. The inclusion of these groups in future research will be crucial for the continued scientific and social progress of aging/dementia and the development of effective interventions.

